# Proprioceptive Rehabilitation of Upper Limb Dysfunction in Movement Disorders: A Clinical Perspective

**DOI:** 10.3389/fnhum.2014.00961

**Published:** 2014-11-25

**Authors:** Giovanni Abbruzzese, Carlo Trompetto, Laura Mori, Elisa Pelosin

**Affiliations:** ^1^Department of Neuroscience, Rehabilitation, Ophthalmology, Genetics and Maternal Child Health, University of Genoa, Genoa, Italy

**Keywords:** proprioception, movement disorders, proprioceptive training, upper limb, sensory feedback

## Abstract

Movement disorders (MDs) are frequently associated with sensory abnormalities. In particular, proprioceptive deficits have been largely documented in both hypokinetic (Parkinson’s disease) and hyperkinetic conditions (dystonia), suggesting a possible role in their pathophysiology. Proprioceptive feedback is a fundamental component of sensorimotor integration allowing effective planning and execution of voluntary movements. Rehabilitation has become an essential element in the management of patients with MDs, and there is a strong rationale to include proprioceptive training in rehabilitation protocols focused on mobility problems of the upper limbs. Proprioceptive training is aimed at improving the integration of proprioceptive signals using “task-intrinsic” or “augmented feedback.” This perspective article reviews the available evidence on the effects of proprioceptive stimulation in improving upper limb mobility in patients with MDs and highlights the emerging innovative approaches targeted to maximizing the benefits of exercise by means of enhanced proprioception.

## Introduction

Movement disorders (MDs) have been traditionally regarded as disorders affecting motor control and resulting from dysfunction of the basal ganglia circuitry. Recently, however, this notion has been largely revised as increasing evidence indicates that MDs are frequently associated with non-motor manifestations (Chaudhuri et al., 2011; Stamelou et al., 2012). Sensory symptoms or abnormalities are frequently observed suggesting a central role of the sensory system in the pathophysiological mechanisms underlying the various MDs (Table 1). In addition, a number of clinical examples suggest that external sensory signals (peripheral sensory feedback) can be used in MDs to compensate the abnormal sensorimotor integration (Abbruzzese and Berardelli, 2003) thus allowing a more effective planning and execution of voluntary movements. Proprioception is generally referred to the conscious awareness of body and limbs as well to the unconscious use of proprioceptive signals for the control of tone and posture (Proske and Gandevia, 2012). Proprioception, therefore, is essential to navigate the environment and drive motor control. Abnormal proprioceptive processing seems to be pivotal in MDs.

**Table 1:** **Somatosensory abnormalities in movement disorders**.

Parkinson’s disease	Increased frequency of pain
	Musculoskeletal, dystonic, radicular, neuropathical, central
	Abnormal proprioceptive (kinesthetic) processing
	Reduced perception of limb position and passive motion
	Impaired sense of heaviness and grip force regulation
	Increased threshold and reduced sensitivity for haptic perception
	Increased somatesthetic temporal discrimination threshold
	Vestibular dysfunction
Dystonia	Increased frequency of pain (musculoskeletal or dystonic)
	Abnormal proprioceptive (kinesthetic) processing
	Reduced perception of passive motion
	Abnormal responses to vibration
	Impaired spatio-temporal discrimination
Chorea	Impaired responses to perturbation
	Pseudo-choreoathetosis
Tics and Tourette syndrome	Discomforting bodily sensations or sensory phenomena (“premonitory urges”)
	Enhanced sensory perception
Restless leg syndrome and akathisia	Discomforting bodily sensations or sensory phenomena (“premonitory urges”)

Currently available treatments (both medical and surgical) for MDs are largely symptomatic. For instance, in Parkinson’s disease (PD), restoring dopaminergic neurotransmission is the essential core of patients’ management. Symptomatic treatments are usually very effective in the early phases, but progressively tend to lose their efficacy with major limitations or side effects (Olanow and Schapira, 2013). Eventually, the development of symptoms that are not responsive to treatment can be observed. Therefore, physiotherapy has become the natural complement of medical/surgical therapies with the aim of promoting motor learning, optimizing the residual functional capacities and compensating for the defective abilities, and thus improving quality of life (Tomlinson et al., 2013).

This perspective article focuses primarily on the rationale and the available evidence of the effects of rehabilitative strategies based on *proprioceptive training* in improving upper limb mobility of patients with MDs (a search of relevant scientific contributions between 1970 and 2014 was performed using PubMed as main database). Furthermore, we highlight the potential importance of innovative interventions targeted to maximizing the benefits of exercise by means of enhanced proprioception.

## Proprioceptive Dysfunction in Movement Disorders

### Parkinson’s disease

Parkinson’s disease patients present consistent abnormalities of proprioceptive integration. The perception of limb position is reduced (Zia et al., 2000), and limb displacements during passive motion are underestimated (Konczak et al., 2007). Additional kinesthetic deficits include the sense of heaviness or weight (Maschke et al., 2006) and grip force regulation (Fellows and Noth, 2004). Such proprioceptive abnormalities, possibly related to changes in the cortical processing of kinesthetic signals (Seiss et al., 2003), may be responsible for abnormal scaling of voluntary movements (Demirci et al., 1997) and make PD patients more dependent on external (mainly visual) cues to initiate or maintain motor activities. Finally, growing evidence indicates that haptic perception (i.e., the ability of recognizing objects through active tactile exploration of their properties such as shape, orientation, and texture) (Gibson, 1966) is commonly impaired in PD. It has been demonstrated that the threshold for ascertaining convex curvature is significantly increased in PD patients as compared to age-matched normal controls (Konczak et al., 2012). It is still controversial whether proprioceptive and haptic changes in PD can be improved by dopaminergic drugs (Li et al., 2010), while subthalamic deep brain stimulation (DBS) partially reverses such abnormalities (Maschke et al., 2005; Aman et al., 2014). Recordings of neuronal activity elicited by passive movements in patients with PD suggest that subthalamic nucleus (STN) DBS may improve kinesthetic acuity by reducing the noise of the signals processed in the STN (Hamani et al., 2004).

### Dystonia

Patients with dystonia often complain of sensory symptoms, though clinical examination is usually normal (Abbruzzese and Berardelli, 2003). The “geste antagoniste” is the sensory phenomenon most frequently observed in dystonia: patients refer that a manipulation of sensory inputs (tactile or proprioceptive feedback) temporarily improves their dystonic postures. The definite mechanisms underlying this phenomenon are still uncertain, but it may be postulated that the sensory tricks act as potential amplifiers of proprioceptive processing (by recruiting additional networks) or induce a motor–sensory adaptation (by updating the *reafference*) (Konczack and Abbruzzese, 2013). Defective kinesthetic perception during passive movements (Putzki et al., 2006) and abnormal vibration-induced illusions of movement (Rome and Grunewald, 1999) have been documented in dystonia. In addition, several observations support the notion that the integration of neck proprioceptive input is impaired in cervical dystonia (Bove et al., 2004, 2007; Muller et al., 2005). Such impaired integration of proprioceptive information with the motor output (and egocentric spatial perception) could be responsible for changes in the internal models of limb dynamics, leading to kinematic abnormalities of reaching movements performed with non-dystonic segments (Pelosin et al., 2009; Marinelli et al., 2011).

### Chorea

The role of proprioceptive abnormalities in choreic patients has been mainly related to problems in standing and walking. However, it should be pointed out that choreic-like dyskinesias may develop because of proprioceptive loss following various nervous lesions. It has been postulated that such “pseudo-choreoathetosis” depends on a failure of processing limb proprioceptive information within the striatum (Sharp et al., 1994).

Altogether, it may be concluded that proprioceptive signals are defective or abnormally processed in both hypokinetic (PD) and hyperkinetic (dystonia, chorea) MDs. Such deficits might lead to difficulties in matching proprioceptive and visual information (for instance, in reaching an object without direct vision of the arm) and contribute to motor impairments in everyday life. It might be postulated, therefore, that rehabilitation of patients with MDs (affecting the upper limbs) could specifically benefit from proprioceptive training in order to increase or modulate the strength of relevant proprioceptive signals. This might translate not only into improved movement kinematics *per se* (i.e., movement speed and spatial accuracy) but also into a better motor performance.

## Proprioceptive Training in Movement Disorders

Proprioceptive training can be defined as the overall set of exercises focused on improving specific components (both conscious and unconscious) directly related to proprioception itself (i.e., joint position sense, force, velocity) or the integration of proprioceptive signals (i.e., movement detection). Within different existing approaches, this type of training is based on the use of “task-intrinsic” feedback (i.e., the sensory–perceptual information that are a natural part of skill performance) or the so-called “augmented feedback” (which refers to adding or enhancing task-intrinsic feedback with an external source) (van Dijk et al., 2005). Although the efficacy of this type of training in stroke is well established (Hayward et al., 2014), evidence regarding its use on upper limb rehabilitation in MDs is poor (Table 2).

**Table 2:** **Summary table of studies on proprioceptive rehabilitation of upper limb dysfunction in movement disorders**.

Citation	Diagnosis	Age (years)	Group	Type of Intervention	Duration of intervention	Results	FU
Deepak and Behari (1999)	Writers’ cramp (WC)	19–62 (range)	WC = 10	EMG biofeedback + writing training	2 months	Improvement (37–93%) in handwriting, alleviation of discomfort, and pain	6 months
Berger et al. (2007)	Writers’ cramp (WC)	28–54 (range)	WC = 5	Biofeedback-based sensorimotor training	5–10 sessions	Substantial improvement of clinical and electromyographic features associated with a significant increase in D2-binding	No
Shumaker (1980)	Parkinson disease (PD)	67.2 (mean)	PD = 20	EMG audio biofeedback + relaxation exercises	15 weeks	Decrease of frontal EMG activity. No significant change of motor task	No
Bienkiewicz et al. (2013)	Parkinson disease (PD)	58–77 (range)	PD = 7; control = 12	Visual cue (artificial sensory guidance)	1 session	Significant improvement of movement time and peak velocity when executing movement in accordance with the information afforded by the point light display	No
Byblow et al. (2003)	Parkinson disease (PD)	65–78 (range)	PD = 12; control = 11	Visual cue (robotic training)	1 session	Improvement in kinesthesis and reaction time performance	No
Del Olmo et al. (2006)	Parkinson disease (PD)	53–68 (range)	PD = 9; control = 5	Auditory cues	20 sessions	Improved regularity of timing with reduction of variability in finger tapping and gait	No
Karnath et al. (2000)	Cervical dystonia (CD)	54	CD = 1	Muscle vibration	1 session	Muscle vibration-induced lengthening of the dystonic neck muscles. Long-term neck muscle vibration was able to reduce head rotation more than short-term vibration	No
Rosenkranz et al. (2008)	Musician Dystonia (MD)	25–31 (range)	MD = 6; WC = 6; control = 12	Muscle vibration	1 session	Proprioceptive training was able to retrain abnormal sensorimotor organization (SMO) toward a more differentiated pattern with potential implications for therapy.	No
King et al. (2009)	Parkinson disease (PD)	33–81 (range)	PD = 40	Whole-body vibration	1 session	Whole-body vibration-induced improvements were seen in UPDRS. Specifically, a significant decrease in rigidity, tremor, and bradykinesia were shown, as well as a significant increase in step length and gait speed.	No
Candia et al. (2005)	Musician Dystonia (MD)	Not reported	MD = 101	Splint + sensory motor retuning training	Not specified	Improved playing performance	3–25 months
Zeuner et al. (2005)	Writers’ cramp (WC)	54.0 ± 8.4 (mean ± SD)	WC = 10	Sensory motor training	20–40 sessions	Significant improvement of dystonia (measured with the Fahn dystonia scale) in all participant. Improvement in writing was recorded in six participants	No
Tinazzi et al. (2006)	Writers’ cramp (WC)	31–42 (range)	WC = 10; control = 12	TENS	16 sessions	RC, DB (versus placebo) trial showing handwriting improvement paralleled by modulation of MEP amplitude in the flexor carpi radialis and the extensor carpi radialis muscle	
Pelosin et al. (2013a)	Focal dystonia (CD and WC)	38–64 (range)	CD = 12; WC = 10	Kinesiotaping	15 days	Randomized, crossover trial showing significant improvement in the subjective sensation of pain (VAS) and reduction of somatosensory temporal discrimination threshold. No improvement of dystonia	No
Ma et al. (2011)	Parkinson disease (PD)	64.77 ± 8.47 (mean ± SD)	PD = 33	VR reproducing ADL at home	1 session	Improvement of movement speed of discrete aiming tasks when participants reached for real stationary objects	No
Ma et al. (2012)	Parkinson disease (PD)	50–78 (range)	PD = 24; control = 24	VR reproducing reaching movements	1 session	VR system providing trunk movement feedback was able to improve speed and coordination of trunk and arm motions during reaching of moving objects	No
Su et al. (2014)	Parkinson disease (PD)	64.76 ± 7.96 (mean ± SD)	PD = 21 control = 21	VR reproducing a ball-catching task + Wii balance board	1 session	The change in performance from slow- to fast-ball conditions was not different between the PD and control groups. The results suggest that raising the speed of virtual moving targets should increase the speed of arm and COP movements for PD patients. Therapists, however, should also be aware that a fast virtual moving target causes the patient to confine the COP excursion to a smaller amplitude	No
Picelli et al. (2014)	Parkinson disease (PD)	Not reported	PD = 10	Robotic training of upper limb	10 sessions	Significant improvement in the nine-hole peg test and in the upper limb section of the Fugl–Meyer scale. Findings were confirmed at 2-week FU evaluation only for the nine-hole peg test. No significant improvement in the UPDRS at both post-treatment and FU evaluations	2 weeks
Herz et al. (2013)	Parkinson disease (PD)	66.7 ± 7.2 (mean ± SD)	PD = 20	Wii console	12 sessions	Significant improvement in NEADL test, PDQ-39 and motor function (UPDRS). FU assessments showed persistent improvement for PDQ-39 and UPDRS scores	1 months
Heremans et al. (2012)	Parkinson disease (PD)	44–67 (range)	PD = 14; control = 14	Motor imagery (MI) + external cues	3 sessions	Visual cues significantly reduced bradykinesia during MI and increased the imagery vividness	No
Pelosin et al. (2013b)	Parkinson disease (PD)	48–77 (range)	PD = 38; control = 14	Action observation (AO) + acoustic cues	1 session	Both AO and cue training increased the spontaneous finger tapping rate in all participants. AO intervention showed a greater effect over time	No

Finally, some more recently developed techniques [virtual reality, gaming consoles, motor imagery (MI) and action observation, robotic rehabilitation) may represent future directions for upper limb rehabilitation in MDs (Table 2).

The early application of biofeedback (BF) technology to neurorehabilitation was in the domain of electromyography (EMG) BF for neuromuscular recovery in individuals with post-stroke hemiparesis. EMG-BF therapy was one of the first approaches used for treating increased muscle tone in generalized and focal (i.e., torticollis) dystonia (Korein and Brudny, 1976; Jahanshahi et al., 1991). Following these preliminary studies, EMG-BF was also applied in writer’s cramp patients, showing positive results in handwriting, reduction of involuntary muscle activity, and pain (Deepak and Behari, 1999). Interestingly, a recent study (Berger et al., 2007) demonstrated that after BF training patients showed, together with an improvement of writing, a restoration of striatal D2-binding receptors, suggesting that a sensory motor training might reorganize the activity of the nigrostriatal dopaminergic system itself. Despite the growing evidence on the use EMG-BF rehabilitation to improve upper limb movements, only a single study was conducted in PD (Shumaker, 1980) and none in Huntington’s disease patients.

Learning-based sensorimotor re-education can also be achieved by different external feedback techniques. The effect of augmented feedback (i.e., external cues) on gait and balance has been extensively studied in PD with a successful application in the clinical practice. However, only few studies investigated the applicability on upper limb dysfunction. Recently, Bienkiewicz et al. (2013) showed the possibility to modulate the speed of reaching movements by a dynamic visual guide suggesting that enhanced perceptual information can partially overcome bradykinesia in PD. These results were comparable with a previous study (Byblow et al., 2003), where it has been demonstrated that the application of visual cues produced a significant improvement of kinesthesis and reaction time performance during bimanual wrist movements. Interestingly, it has been demonstrated (Del Olmo et al., 2006) that a 4-week training based on auditory cues was able to induce a reduction of temporal variability, not only in walking performance but also in other repetitive movements such as finger tapping, suggesting the possibility of motor learning transfer among similar movements regulated by the same neural networks. Although external cues have been applied to investigate possible modifications in movement planning (i.e., externally triggered versus self-initiated movements) in MDs other than PD, no data are reported for their application on upper limb rehabilitation.

Another consolidated approach in neurorehabilitation to induce proprioceptive stimulation is represented by muscle vibration (for a review, see Murillo et al., 2014). A few pilot studies investigated the use of focal vibration as a proprioceptive facilitator to promote motor control in functional activities of patients with MDs. For example, in a single case study (torticollis), long-term neck muscle vibration was associated with improvements in head and trunk position (Karnath et al., 2000). More specifically, Rosenkranz et al. (2008) used a proprioceptive training (repeated cycles of fingers muscle vibration) in pianists with musician’s dystonia. Focal muscle vibration was able to restore sensorimotor organization to the pattern observed in healthy pianists. More importantly, task-specific motor control improved objectively and was significantly correlated to the degree of sensorimotor reorganization.

No study investigated the possible effect of focal vibration on upper limb function in patients with PD. However, an improvement of gait was reported in these patients following the application of vibration to lower limb muscles (Novak and Novak, 2006; De Nunzio et al., 2010) and changes were attributed to the enhancement of proprioceptive feedback. On the other hand, King et al. (2009) investigated the effect of “whole-body” vibration (sound waves) in PD showing significant improvements in outcome measure including rigidity, tremor, and motor function (improved speed on the grooved pegboard task).

Furthermore, alternative rehabilitative strategies aimed to modulate sensory processing and proprioceptive feedback by means of sensory retraining or retuning and learning-based sensorimotor re-education. These approaches (Braille reading, discriminative exercises), alone or associated with selective splinting of dystonic muscles, have been used successfully in patients with focal hand dystonia (writer’s cramp, musician’s cramp) although the benefit duration was generally short-lasting (Candia et al., 2005; Zeuner et al., 2005). Transcutaneous electrical nerve stimulation (TENS) was also used to activate muscle afferents in order to modulate corticomotoneuronal excitability and restore a balance in upper limb muscles of patients with focal hand dystonia (Tinazzi et al., 2006).

Finally, kinesiotaping was recently proposed as a tool for enhancing proprioception. We demonstrated (Pelosin et al., 2013a) that treatment with kinesiotaping might reduce pain and modulate sensory function in patients with focal cervical or hand dystonia, although no improvement of the motor pattern was observed. Kinesiotaping was not investigated so far in upper limb dysfunction of PD patients.

## Future Directions for Upper Limb Proprioceptive Rehabilitation in MDs

Over the past decades, an important development has occurred in the field of MDs rehabilitation. Together with the predominant focus on finding new approaches designed to the recovery of impaired movements (especially in PD), concomitant efforts have been dedicated to search specific strategies for improving motor learning. In particular, a great emphasis was placed on the use of sensory feedback in promoting neuroplasticity (Nudo, 2006). The establishment of the concept of plasticity-based functional training gave rise to the idea of applying new technologies to the rehabilitation field. In this section, innovative approaches based on proprioceptive training will be briefly described.

Virtual reality (VR) can be defined as a “high-end-computer interface that involves real time simulation and interactions through multiple sensorial channels” (Burdea, 2003). VR is becoming more and more widespread in the rehabilitation of patients with neurological disorders. The clinical application of VR is based on the interaction of the person with a virtual environment with the aim to promote motor learning taking advantage of enhanced perceptions (visual, auditory, and haptic inputs). In VR, the external feedback is driven from the environment and can be compared with internal proprioceptive sensations as well as with the acquired knowledge of the performance. VR systems have been successfully applied in the recovery of upper limb function especially in patients with stroke (for a review, see Laver et al., 2011). However, in the last decade, this technology has been implemented also in PD patients. Although most of the studies were focused on improving balance (Yen et al., 2011) and gait (Mirelman et al., 2011), the evidence supporting the efficacy on upper limb function is increasing. Ma et al. (2011) designed a randomized controlled trial to verify the effect of VR training on functional reaching movements in a cohort of subjects with PD. They showed that, after a short period of training, the VR program improved the movement speed of discrete aiming tasks when participants reached for real stationary objects, while the transfer effect was minimal when reaching for real moving objects. Further, they also demonstrated (Ma et al., 2012) that combining VR system with trunk movement feedback was able to improve the speed and coordination of trunk and arm motions during reaching of moving objects. Finally, in a recent study, it has been showed that training based on the manipulation of the speed of virtual moving targets improved arm movement and standing postural control in parkinsonian patients (Su et al., 2014). Despite these promising results, to our knowledge, no study investigated the possible effect of VR application on upper limb function in dystonia, Huntington’s disease, or other MDs.

In addition to custom VR systems, commercial gaming consoles (i.e., Nintendo Wii™, Xbox Kinect™, PlayStation™) have been also introduced as new tools for rehabilitation. These technologies have been considered useful in physiotherapy not only because they are low cost and can be used at home to promote physical activity but also because these systems are able to generate visual/auditory stimuli as well as different types of augmented proprioceptive feedback (for instance, vibratory stimulations). Although preliminary positive results were obtained in upper limb rehabilitation of patients with various neurological diseases (Winkels et al., 2013; Pietrzak et al., 2014), gaming consoles have been mainly used in order to improve balance and increase physical activity in PD patients (Esculier et al., 2012). However, in a recent study (Herz et al., 2013), it has been demonstrated that after 4 weeks of Wii-training PD patients significantly improved motor functions (UPDRS), daily living activities, and quality of life (PDQ-39). The results of the latter study suggest the possibility to apply this technology also for recovery of upper limb movements. To date, however, no study has been published in any MDs other than PD.

Robotic rehabilitation has grown rapidly in recent years, and it represents a promising novel technology for recovery of motor function in patients with neurological diseases. The most important advantage of using robot technologies is the possibility to deliver high-dosage and high-intensity training. Recent reviews have shown that robot-assisted arm training can be considered an innovative approach in order to improve upper limb function in stroke (Mehrholz et al., 2012; Wolf et al., 2014) and cerebral palsy (Fasoli et al., 2012). A recent study (Picelli et al., 2014) showed that robot-assisted arm training was able to improve upper limb function in 10 patients with PD.

Finally, also MI and action observation therapy (AOT) should be considered promising approaches for improving proprioceptive deficits in MDs. MI is described as the mental representation (to feel or see) of actions in the absence of overt movement. MI is a highly complex mental process that is thought to primarily involve sensory, perceptual, and affective brain areas by means of generating internal feedback. Specifically, internal MI (also known as kinesthetic or first-person imagery) results to be superior to external MI (also known as third-person visual imagery) for improving motor skills by enhancing proprioceptive signals normally generated during movements. AOT is a technique based on the activation of the mirror neuron system (Jacoboni and Mazziotta, 2007), consisting in the observation of different actions combined with the repetition of the observed actions. Both MI and AO share the common mechanism of facilitating the subsequent movement execution by directly matching the imagined or observed action with the internal representation of that action thus potentially enhancing the learning of new tasks and improving the performance of impaired tasks (Mirelman et al., 2013). The use of these approaches is slowly emerging and there is evidence suggesting their potential applicability to rehabilitation of patients with MDs. Indeed, it has been shown that combining visual cues with MI can improve upper limb movement execution in PD patients (Heremans et al., 2012). Similarly, we demonstrated a reduction of bradykinesia of finger movements by a single session of AOT (Pelosin et al., 2013b). These innovative approaches may have important clinical implications for training PD patients in everyday tasks and they may be extended also to patients with other MDs. However, since a close relationship exists between MI and AO ability and their clinical effectiveness, a thorough screening of patients’ ability is necessary before considering MI or AO as potential tools to enhance rehabilitative protocols.

## Conclusion

It is largely established that proprioceptive sensory inputs are fundamental in the generation and coordination of movements and that their abnormalities may underlie many hypokinetic and hyperkinetic disorders. Proprioceptive rehabilitation is aimed at improving or enhancing the perception of proprioceptive signals and their central integration thus possibly compensating the impaired “gating” function of the basal ganglia.

We presented an overview of proprioceptive rehabilitation in MDs showing that proprioceptive training is a promising tool for improving motor control in MDs. However, we underlined that there is an important gap in the literature concerning upper limb rehabilitation in patients with PD and other MDs. Further studies should be planned in order to evaluate the potential role of all the modalities of proprioceptive training and their possible learning effect by re-examining large study populations after a follow-up period.

## Conflict of Interest Statement

The authors declare that the research was conducted in the absence of any commercial or financial relationships that could be construed as a potential conflict of interest.
